# Psychotropic Drug Use in São Paulo, Brazil – An Epidemiological Survey

**DOI:** 10.1371/journal.pone.0135059

**Published:** 2015-08-07

**Authors:** Maria Ines Quintana, Sergio Baxter Andreoli, Marcela Poctich Peluffo, Wagner Silva Ribeiro, Marcelo M. Feijo, Rodrigo Affonseca Bressan, Evandro S. F. Coutinho, Jair de Jesus Mari

**Affiliations:** 1 Universidade Federal de São Paulo—Escola Paulista de Medicina, Psychiatry Department, São Paulo, Brazil; 2 Universidade Catolica de Santos, Santos, Brazil; 3 Escola Nacional de Saude Publica—Fundação Oswaldo Cruz, Rio de Janeiro, Brazil; Hospital Universitário, Centro de Pesquisa Clínica e Epidemiológica, BRAZIL

## Abstract

**Objective:**

To estimate the prevalence of one month psychotropic drug use in São Paulo, Brazil, and to assess the gap treatment between the presence of mental disorders and psychotropic drug users.

**Method:**

A probabilistic sample of non-institutionalized individuals from the general population of São Paulo (n = 2336; turnout: 84.5%) who were 15 years or older were interviewed by a trained research staff, applying the Composite International Diagnostic Interview 2.1 (CIDI WHO) (depression, anxiety-phobia, OCD\PTSD, alcoholism sections), and an inventory investigating psychotropic drug use during the 12-month and one-month periods immediately preceding the interview. Logistic models were fitted to investigate associations between psychotropic drug use as well as socio-demographic and clinical variables.

**Results:**

The one month prevalence of psychotropic drug use in São Paulo was 5.89%, the most commonly used drugs were antidepressants (3.15%) and tranquilizers (2.67%). A higher consumption of psychotropic drugs (overall, antidepressants and tranquilizers) was observed among women (OR:2.42), older individuals (OR:1.04), individuals with higher levels of formal education (1.06), and individuals with a family (OR:2.29) or personal history of mental illness (OR:3.27). The main psychotropic drug prescribers were psychiatrists (41%), followed by general practitioners (30%); 60% of psychotropic drugs were obtained through a government-run dispensing program. Most individuals who obtained a positive diagnosis on the CIDI 2.1 during the previous month were not using psychotropic medication (85%). Among individuals with a diagnosis of moderate to severe depression, 67.5% were not on any pharmacological treatment.

**Conclusion:**

There is a change in the type of psychotropic more often used in São Paulo, from benzodiazepines to antidepressants, this event is observed in different cultures. The prevalence of use is similar to other developing countries. Most of the patients presenting a psychiatric illness in the month prior to testing were not receiving any sort of psychiatric medication. This may be explained by a failure to identify cases in primary care, which could be improved (and access to treatment could be facilitated) if professionals received more specialized training in managing cases with mental health problems.

## Introduction

In the last 40 years, several factors influence the consumption of psychotropic drugs in Brazil. Among them the consistent supply of new psychotropic medications on the market, the prohibition to sell “antidistonics” substances (benzodiazepines associated with antispasmodics) over-the-hold in the late 80 [1]; the increased psychotropic applicability to clinical areas [2, 3] and more recently in the late 90 the implementation of the Estratégia de Saúde da Família (ESF; Family Health Strategy Program). During the 1970s the annual consumption of psychotropic drugs where 12.12% in the city of São Paulo [4], which decreased to 10.2% 1980s [1] and to 7.1% in 2002 [5].

For European countries such as France, Germany, Italy and United Kingdom, Ohayan et al., refer 6.4% montly prevalence of psychotropic consumption (data collected between 1993–1997) [6]. With a similar rate was Spain (6.9%) [7] and Canada (7.2%) [8]. Data from the National Health and Nutrition Examination Surveys (NHANES) showed an increase of monthly consumption of psychotropic drugs among Americans from 6.1% in 1988–1994 (NHANES III) to 11.1% in 1999–2002 (NHANES 1999–2002) [9].

Studies conducted at the beginning of this century revealed a shift from benzodiazepine to antidepressant use [10, 11], and particularly serotonin-specific reuptake inhibitors (SSRIs). In the USA, the increase of antidepressant use was 5.9% in 1996 to 8.1% in 2001, and SSRIs and other new forms of antidepressants were responsible for 80% of this increase [10].

As described by several authors of studies conducted across different cultures, psychotropic drug consumption tends to be more common among women and increases with age and socioeconomic status [1, 4, 8, 12–14]. There is a significant gap between the presence of a psychiatric diagnosis and the use of psychotropic drugs (treatment gap). Treatment gap is high in most studies, independently of economic status and culture. In Brazil, it varies from 80% in Rio de Janeiro [15] to 85% in São Paulo [5], 81% in Canada [8], 84% in the UK [16], and 67% in a European multicenter study [17]. Despite an increase in the number of public health networks, specialists and family physicians in recent years, several references in the literature assert that a large number of individuals with mental disorders remains without drug treatment. Without considering the inherent barriers [18] of treatment seak to the public or private health systems, some hypotheses attempting to explain this observation are that: 1) individuals do not seek help or do not recognize their symptoms; and 2) professionals do not recognize psychiatric symptoms [15].

Faced with these issues, one must understand what the barriers to treatment are, as well as the stigma associated with searching for solutions to mental problems. The aim of this study was to evaluate the prevalence of psychotropic drug use and its association with the presence of mental disorders (DSM IV) assessed for the month prior to participant interviews, based on epidemiological data from a representative population sample from the city of São Paulo, Brazil. In addition, we evaluated a) users’ sociodemographic profile; b) how they obtained medications; and c) who prescribed them.

## Methods

### Settings and study design

The present study was based on data from a larger project entitled “Violence and post-traumatic stress disorder in São Paulo and Rio de Janeiro, Brazil”. The detailed study protocol is available online [19]. As part of that study, a population-based cross-sectional survey was conducted in São Paulo, Brazil, between February and June of 2007. The sample was representative of the population and included individuals aged 15–75 years. The city is divided into 96 administrative sectors, which were stratified into seven strata according to homicide rate (index of violence) [20]; then, 4 to 18 sectors were randomly selected according to the size of the population in each stratum. In order to increase the likelihood of identifying post-traumatic stress disorder cases, the three strata with the highest homicide rates were oversampled. Finally, 30 households per census sector were randomly selected within each stratum and one resident in each selected household was randomly selected to be interviewed according to Kish’s method [21]. The study design was submitted and approved by the ethics committee of the Federal University of São Paulo (process n. 1369/04). Written informed consent was given for all participants for their clinical records to be used in this study.

### Measurements

Socio-demographic variables were collected using a questionnaire specifically designed for the study. The variables of interest in this analysis were gender, age, marital status, formal education, income, ethnic group, history of migration, and family history of mental illness; the clinical variable was psychiatric diagnosis during the month prior to the study. We used the 2.1 version of the Composite International Diagnostic Interview (CIDI 2.1) to assess mental disorders (anxiety, depressive disorders, and alcohol misuse/dependence). The CIDI 2.1 is a standardized and fully structured interview that provides psychiatric diagnoses through computerized algorithms according to the Diagnostic and Statistical Manual of the American Psychiatric Association, 4th edition (DSMIV) and the International Classification of Diseases (ICD10). The Brazilian Portuguese version of the CIDI 2.1 has been previously validated and adapted to Brazil’s social and cultural context [22–24].

Participants were asked the following question: ‘‘Have you taken any medication for a nervous breakdown, emotional, psychological, or psychiatric problem, or seizures in the last year?” Individuals who provided a positive answer were subsequently questioned about drug use during the previous month (i.e., the type of medication, who prescribed it, and where the medication was obtained). Information cards were given to each respondent containing information about generic and trade psychotropic drug names available on the Brazilian market, medical specialties, health professionals, and ways to obtain medications. Psychotropic drugs were primarily classified by their pharmacological group, taking into account their main clinical indications. The change of psychotropic drug use in the city of São Paulo was assessed by comparing the data for the previous month with previous epidemiological studies using similar methods of data collection.

### Procedures

Face-to-face interviews were performed by a team of lay interviewers working for the Brazilian Institute of Public Opinion and Statistics (IBOPE), which is one of the largest Brazilian independent research institutes. The interviewers were trained to apply the CIDI 2.1 by researchers from an official CIDI\WHO\UNIFESP Training Center. The interviewers were also trained to apply the full set of questionnaires used in the study by this study’s authors. To optimize response rates, interviewers contacted the selected households up to ten times. Households that did not respond after ten attempts were excluded. Usually, interviews were completed in one visit, but occasionally, a second visit was necessary.

### Statistical analysis

Statistical analyses were conducted using the Statistical Package for the Social Sciences 19 (SPSS) and STATA complex sample analysis. Data were weighted to account for oversampling of people living in the most violent strata and for differential probability of selection, in order to allow for the estimation of population-level prevalence estimate. The prevalence of one year and one month psychotropic drug use and mental disorders was estimated using complex sample surveys, linearization to estimate variances and weighted data.

Logistic models using the enter method of entry were fitted to investigate associations between use of psychotropic drugs, socio-demographic and clinical variables. The first model included variables with p <0.10 from the univariate analysis. In the second stage, variables whose p-values were larger than 0.05 were dropped from the model, except for the variable ‘education’.

## Results

From 3000 households initially selected, a total of 2536 individuals were interviewed, with a loss of 15.5%. Reasons for loss included: an inability to establish contact after ten visits, refusal to participate or to sign the consent term, and an inability to gain access to participants’ homes. The sample was predominantly female (58%), with a mean age of 39.5 years (with no age difference across genders) and 47% of respondents were aged 20–39 years. Most participants had migrated to the city of São Paulo, were working at the time of the study, were Catholic and Caucasian and had 11 or more years of schooling. Half of the individuals interviewed reported a monthly per capita income equivalent to US$ 255.10, which was the minimum wage in 2007, the year the study was conducted (Table 1).

**Table 1:** Sample distribution and prevalence of psychotropic drug use during the previous 30 days by sociodemographic characteristics (total sample = 2536; one-month psychotropic use = 145).

Variable	total sample % (CI 95%)	one-month psychotropic use% (CI 95%)
Psychotropic use		5.5 (4.8–7.2)
Gender		
Male	43.2 (39.5–44.3)	2.9 (2.1–4.0)
Female	56.8 (55.6–60.5)	7.5 (6.2–9.0)
Age		
15–39 years	59.6 (52.7–57.6)	2.9 (2.2–3.9)
40–59 years	29.8 (30.5–35.2)	10.3 (8.2–12.9)
> 60 years	10.5 (10.5–13.7)	6.7 (4.2–10.5)
Marital Status		
married/living with someone	57.8 (54.5–59.3)	5.3 (4.3–6.6)
single/not living with someone	42.1 (40.7–45.5)	5.8 (4.5–7.4)
Employed		
No	38.5 (37.3–42.1)	8.0 (6.5–9.8)
Yes	61.5 (57.8–62.7)	4.0 (3.1–5.0)
Years of schooling		
Illiterate	3.5 (2.2–3.8)	6.7 (3.2–13.4)
< 8 years	47.6 (40.3–45.0)	5.6 (4.4–6.9)
9–11 years	35.6 (34.3–39.0)	4.7 (3.4–6.3)
> 12 years	13.4 (15.9–19.9)	7.4 (4.7–11.4)
Place of origin		
SP	45.4 (47.5–52.4)	5.9 (4.8–7.3)
Other	54.6 (47.6–52.5)	5.0 (3.8–6.6)
Religion		
Catholic	60.5 (59.7–64.4)	5.1 (4.1–6.3)
Evangelical	24.4 (20.7–24.8)	5.8 (4.2–8.0)
Spiritualist	4.2 (4.2–6.4)	10.5 (5.7–18.4)
Other	10.5 (8.7–11.6)	4.5 (2.5–7.8)
Ethnicity		
Caucasian	44.1 (48.5–53.4)	6.8 (5.4–8.5)
black/mixed	52.0 (42.4–47.2)	4.8 (3.8–6.0)
Other	3.9 (3.3–5.4)	1.0 (0.1–7.0)
Family Income		
< 159 US$	30.0 (21.3–25.3)	5.1 (3.7–7.1)
160–272 US$	24.3 (19.7–23.8)	5.1 (3.6–7.3)
273–476 US$	23.3 (24.2–28.8)	5.2 (3.6–7.4)
>477 US$	22.3 (26.3–31.1)	7.3 (5.2–10.1)
Family history of mental illness		
No	77.4 (75.7–79.8)	3.8 (3.01–4.74)
Yes	22.6 (20.2–24.3)	11.2 (8.88–14.10)
CIDI lifetime diagnosis		
No	55.4 (53.4–58.3)	2.2 (1.6–3.1)
Yes	44.6 (41.7–46.6)	9.6 (8.0–11.6)

The monthly prevalence of psychotropic drug use was 5.52%, with antidepressants as the most widely used (3.15%), followed by tranquilizers (2.67%), antipsychotics (0.67%), mood stabilizers (0.53%) and barbiturates (0.52%). Antidepressant, tranquilizer, anorectic and overall drug use was higher in women than men, while there was no difference in use between genders for the other drug categories (Table 2). During the month prior to the interview, 60% of respondents used monotherapy, 26% used two different psychotropic drugs and 14% used three or more psychotropic drugs simultaneously.

**Table 2:** One-month prevalence of use of different psychotropic drugs in São Paulo by gender (n = 2536).

	man (n = 1096)			female (n = 1440)			total (n = 2536)		
	N	%	IC95%	N	%	IC95%	n	%	IC95%
All psychotropic drugs	32	3.2*	1.8–4.6	113	7.8	6.1–9.5	145	5.5	4.8–7.2
Antidepressants^1^	12	1.2*	0.3–2.0	64	4.5	3.2–5.9	76	3.1	2.29–4.0
1ª generation	6	0.4	0.04–0.7	27	1.7	0.9–2.6	33	1.2	0.68–1.7
2ª generation	8	1.0	0.2–1.9	44	3.5	2.3–4.7	52	2.4	1.66–3.2
Anorectic drugs^2^	0	0*	0	6	0.7	0.1–1.3	6	0.4	0.04–0.8
Mood stabilizers^3^	5	0.7	0.04–1.4	6	0.4	0.05–0.7	11	0.5	0.18–0.9
Tranquilizers^4^	8	1.0*	0.2–1.8	50	3.9	2.6–5.2	58	2.7	1.85–3.5
Antipsychotic drugs^5^	5	0.5	0–1.0	11	0.8	0.2–1.37	16	0.7	0.28–1.1
1ª generation	3	0.4	0–0.9	10	0.7	0.2–1.2	13	0.6	0.20–0.9
2ª generation	2	0.1	0–0.2	2	0.3	0–0.7	4	0.2	0–0.4
Others^6^	5	0.3	0.03–0.5	10	0.8	0.2–1.4	15	0.6	0.2–0.9
Anticholinergics^7^	1	0.1	0–0.1	3	0.2	0–0.4	4	0.1	0–0.2
Barbiturates^8^	6	0.5	0–1.0	7	0.5	0.06–1.0	13	0.5	0.16–0.9
Alcoholism treatment^9^	1	0.05	0–0.2	2	0.1	0–0.2	3	0.1	0–0.1
Hypnotics^10^	1	0.05	0–0.1	2	0.2	0–0.8	3	0.1	0–0.3
Attention Deficit^11^	0	0*	0	1	0.2	0–0.5	1	0.1	0–0.3

* Signficantly different from women (p<0.001)

^1^Antidepressants: escitalopran, sertraline, bupropion, paroxetine, citalopram, fluoxetine, venlafaxine, duloxetine, fluvoxamine, amitriptyline, imipramine, nortriptyline, clomipramine, trazodone, tianeptine, reboxetine;

^2^Anorectics: sibutramine, mazindol, fluramina hydrochloride femproporex, diethylpropion hydrochloride, amfepramone;

^3^Mood stabilizers: gabapentin, carbamazepine, lithium carbonate, divalproex sodium, lamotrigine, topiramate, oxazepan;

^4^Tranquilizers: clorazepate, diazepam, alprazolam, bromazepam, clonazepam, chlordiazepoxide, lorazepam, clobazam, cloxazolam;

^5^Antipsychotics: aripiprazole, quetiapine, ziprasidone, risperidone, clozapine, pipotiazine, penfluridol, zuclopenthixol, fluphenazine, thioridazine, trifluperazina, chlorpromazine, amisulpride, haloperidol, tianeptine;

^6^Other: buspirone others;

^7^Anticholinergics: biperiden hydrochloride, acamprosate;

^8^Barbiturates, phenytoin, phenobarbital;

^9^Alcoholism: dissulfuran, naltrexone;

^10^Hypnotics: nitrazepam, zolpidem, triazolam, flunitrazepam, midazolam maleate;

^11^Attention Deficit: methylphenidate, atomoxetine.

The annual prevalence of psychotropic drug use was 8.79%: 12.5% for women and 5% for men. Antidepressants (4.72%) were the most commonly used (and were four times more prevalent among women), followed by tranquilizers (4.20%; which were twice as frequent among women as among men). All other drugs had a prevalence below 1% (Table 3).

**Table 3:** One-year prevalence of psychotropic drug use in São Paulo by gender.

	Male (n = 1096)			female (n = 1440)			Total (n = 2536)		
	N	%	IC95%	n	%	IC95%	n	%	IC95%
All psychotropic drugs	46	5.0*	3.4–6.8	177	12.5	10.3–14.6	223	8.8	8.0–10.9
Antidepressants^1^	16	1.9*	0.8–3.0	91	6.7	5.1–8.4	107	4.7	3.6–5.8
1ª generation	8	1.4*	0.4–2.4	37	5.3	3.8–6.8	45	3.7	2.7–4.7
2ª generation	10	0.7	0.1–1.3	67	2.5	1.5–3.6	77	1.8	1.1–2.4
Anorectics^2^	0	-*	-	18	1.6	0.7–2.6	18	1.0	0.4–1.5
Mood stabilizers^3^	6	0.7	0.04–1.4	13	0.7	0.2–1.2	19	0.7	0.3–1.1
Tranquilizers^4^	20	2.4*	1.2–3.6	82	5.5*	4.04–6.9	102	4.2	3.2–5.2
Antipsychotics^5^	7	0.7	0.1–1.2	15	1.2	0.5–1.9	22	1.0	0.5–1.4
1ª generation	3	0.4	0–0.9	13	0.9	0.3–1.5	16	0.7	0.3–1.1
2ª generation	4	0.3	0–0.6	3	0.4	0–0.9	7	0.4	0.05–0.7
Others^6^	20	0.3	0.1–0.5	56	1.2	0.49–2.0	76	0.8	0.4–1.3
Anticholinergics^7^	1	0.05	0–0.1	3	0.2	0–0.4	4	0.1	0–0.2
Barbiturates^8^	6	0.5	0–1.0	7	0.5	0.1–1.0	13	0.5	0.2–0.9
Alcoholism^9^	2	0.2	0–0.5	3	0.1	0–0.2	5	0.1	0–0.3
Hypnotics^10^	1	0.05	0–0.1	5	0.4	0–0.8	6	0.3	0–0.5
Attention Deficit^11^	0	-*		1	0.2	0–0.5	1	0.1	0–0.3

*Signficantly different from women (p<0.001)

^1^Antidepressants: escitalopran, sertraline, bupropion, paroxetine, citalopram, fluoxetine, venlafaxine, duloxetine, fluvoxamine, amitriptyline, imipramine, nortriptyline, clomipramine, trazodone, tianeptine, reboxetine;

^2^Anorectics: sibutramine, mazindol, fluramina hydrochloride femproporex, diethylpropion hydrochloride, amfepramone;

^3^Mood stabilizers: gabapentin, carbamazepine, lithium carbonate, divalproex sodium, lamotrigine, topiramate, oxazepan;

^4^Tranquilizers: clorazepate, diazepam, alprazolam, bromazepam, clonazepam, chlordiazepoxide, lorazepam, clobazam, cloxazolam;

^5^Antipsychotics: aripiprazole, quetiapine, ziprasidone, risperidone, clozapine, pipotiazine, penfluridol, zuclopenthixol, fluphenazine, thioridazine, trifluperazina, chlorpromazine, amisulpride, haloperidol, tianeptine;

^6^Other: buspirone others;

^7^Anticholinergics: biperiden hydrochloride, acamprosate;

^8^Barbiturates, phenytoin, phenobarbital;

^9^Alcoholism: dissulfuran, naltrexone;

^10^Hypnotics: nitrazepam, zolpidem, triazolam, flunitrazepam, midazolam maleate;

^11^Attention Deficit: methylphenidate, atomoxetine

The main prescribers were psychiatrists (41%), followed by general practitioners (30.2%), neurologists (14.4%), cardiologists (5.9%), and other medical specialists (5.4%). In all classes of psychotropic drugs surveyed, psychiatrists were the main prescribers, followed by general practitioners. The rate of "prescriptions" for psychotropic drugs made by non-physicians (e.g., friends, family, religious leaders, pharmacists) was 12.2%. For drug use among cases of moderate/severe depression, 17.3% of prescribers were psychiatrists, 14.3% were general practitioners, 6.1% were other types of physicians and 5.1% were non-physicians. Further, 60% of psychotropic drugs were obtained through government dispensing programs, especially antipsychotics, which in 56% of cases were obtained for free. Antidepressants showed a similar distribution among government dispensing programs (46%) and purchased with own resources (47%). Taking into account antidepressant 54% of tricyclic were obtained from the government and 41% were bought by the respondent (p = 0.007). The reverse happened with SSRIs and other antidepressants in which 24% was obtained by the government and 70% was bought by own resources (p = .000). Unlike tranquilizers in 60% of cases were purchased with own funds and 30% provided by the government.

In the logistic regression model, the variables showing a greater association with monthly psychotropic drug use were: female gender (OR: 2.42), increasing age (OR: 1.04), higher level of formal education (OR: 1.06), a history of mental illness in the family (OR: 2.29) and a personal history of mental illness (OR: 3.27) (Table 4).

**Table 4:** Parameter estimates for the simultaneous effects of sex, age, education, marital status, race, religion, family income, being a case in CIDI 2.1 (one-month), family history of mental illness and one-month psychotropic consumption (n = 2536) in the city of São Paulo.

	OR	P	CI 95%	
			Lower	Upper
Gender	2.42	0.00	1.69	3.48
Age	1.04	0.00	1.03	1.05
Education (years)	1.06	0.00	1.02	1.11
Marital status				
married	Reference			
never married	0.64	0.19	0.33	1.25
Separated/divorced	1.39	0.15	0.89	2.19
widowed	1.06	0.75	0.73	1.56
Family mental illness	2.29	0.00	1.68	3.13
Family income	0.62			
< 160	Reference			
160–272 US$	1.03	0.87	0.68	1.56
273–476 US$	0.79	0.94	0.51	1.25
>477 US$	1.02	0.00	0.64	1.61
CIDI one month	3.27	0.00	2.39	4.48
Constant	0.004			

We found that 85% of individuals with a psychiatric diagnosis through the CIDI 2.1 were not using any psychotropic drugs. Among individuals with depression, only 19.5% were using antidepressants and 26.8% were using another psychotropic drug. Among cases with moderate and severe depression, there was an increased number of individuals making use of any psychotropic drug (32.5%) as well as antidepressants specifically (26%), but the treatment gap was still 67.5%. The treatment gap for phobic anxiety disorders was 86%, 76% for obsessive compulsive disorders, and 74% for PTSD (Table 5).

**Table 5:** Relationship between individuals with a psychiatric diagnosis on the CIDI 2.1 during the previous month, psychotropic drug use, and number of individuals with a psychiatric diagnosis during the previous month who did not receive psychotropic drugs.

	N	tranquilizers	Antidepressants	GAP
Depression*	149	12.8 (19)	19.5 (29)	73.2 (109)
Mild	64	14.5 (9)	14.5 (9)	75.8 (47)
Moderate	50	14.0 (7)	34.0 (17)	60.0 (30)
Severe	27	11.1 (3)	11.1 (3)	81.5 (22)
Dysthymia	13	0	7.7 (1)	92.3 (12)
Depression mod/sev**	77	13.0 (10)	26.0 (20)	67.5 (52)
Transphobic-anxious	306	8.2 (25)	9.8 (30)	85.6 (262)
OCD	81	12.3 (10)	18.5 (15)	76.5 (62)
TEP	101	9.9 (10)	18.8 (19)	74.3 (75)
Any of the above	468	7.1 (33)	10.5 (49)	85.3 (399)

*sum of all three degrees of severity and dysthymia;

** only cases with a diagnosis of moderate or severe depression; atd: antidepressants; GAP: individuals with a positive diagnosis in the previous month that do not use psychotropic drugs.

## Discussion

During 2007, 5.5% of the persons aged 15–75 in São Paulo were using a psychotropic drug in the past month. This was not a significant change from earlier years. Antidepressants (3.15%) and tranquilizers (2.67%) where the psychotropics most commonly used. We found a greater rate of psychotropic drug use among females, as well as individuals who were older, had a higher level of formal education, and a family or personal history of mental illness. Among individuals taking any of these medications, most used monotherapy. The main prescribers were psychiatrists, followed by general practitioners. Finally, 60% of psychotropic drugs were obtained through government dispensing programs. The number of individuals with a positive DSMIV diagnosis who were not taking psychotropic drug treatment (treatment gap) was 85%: 67.5% for moderate/sever e depressive disorder, 86% for-phobic anxiety disorders, 76% for OCD and 74% for PTSD.

The monthly prevalence of psychotropic drug use in São Paulo was similar to that reported for Rio de Janeiro (6.5%) during the same period [15]. The differences observed between these two cities concern mostly the type of drugs used, individuals' income, how drugs are obtained, and the prescribers. While people in Rio de Janeiro used more antidepressants, followed by anorexigenics, in São Paulo, the most common drugs were antidepressants, followed by benzodiazepines. In Rio de Janeiro, higher income individuals used more psychotropic drugs, while this pattern was not observed in São Paulo. A likely explanation for this difference may be how drugs were obtained: in São Paulo, 60% of respondents obtained psychotropic drugs from the government for free, while this was true for only 13% of respondents in Rio de Janeiro.

The use of psychotropic drugs in the past month in São Paulo was found to be similar to studies from Chile (6.4%) [25] and the United States (5.5%) [12], higher than that reported in England (3.5%) (16) and lower than that reported in Canada (7.2%) [8] and Australia (10.6%) [26]. In Brazil, in 2001, in São Paulo in 3-day period the consumption of psychotropic drugs was 13% [27], while in 2003 in a period of two week period the prevalence was 10% in the southern of the country [27] and 5% in a study that evaluated different regions of Brazil [28]. It is relevant to point out that the health system in the city studied in the south is better develop than the rest of Brazil, this could explain the higher prevalence.

The social and demographic characteristics of psychotropic drug users in São Paulo found in the current study have been [1, 4, 9, 15, 27, 29, 30] previously reported in the literature, such as higher drug use among females, older individuals, those with higher education levels, and those with a family history and positive diagnosis for mental illness [1, 4, 8–10, 14, 15, 27, 29–31].

The influence of economic status is rather controversial across studies. Some authors have reported an increase in psychotropic drug use among lower income individuals [11, 12, 32], while others have found increased use among higher income individuals [1, 4, 5, 8, 14, 27, 30, 33]. In this study, we found higher psychotropic drug use among individuals with higher income, but this difference was not statistically significant. The effect of income was lower in São Paulo, and most of the individuals interviewed (60%) had access to free medication. Thus, equity of access to medication may inhibit the effect of the inverse care law [34]. Contributing to this finding is the fact that most prescriptions were made by psychiatrists, which shows that patients, in São Paulo are having more access to specialists, effect comproved by Blay et. all. that found that 49% of the population studied in São Paulo in 2002, had access to psychotropic drugs prescribed by psychiatrists [5] It is noteworthy that access to psychiatrists does not mean better quality of mental health treatment.

When we look back on previous studies conducted in São Paulo, we observed that the annual prevalence of psychotropic drug use has remained stable over the last three decades in the city, with a mild decreasing trend. The prevalence of overall drug consumption in São Paulo in the 70s was 12.9% [4], decreasing to 10.6% in the 80s [1], 7% [5] in 2002 and then to 8.8% in 2007. Comparing to results from the WHO World Mental Health Surveys vary from, our rates are similar in Spain (16%) [35], lower than France (21%) [36], Belgium (19%) [37], but higher than Israel (7%) [38], Canada (7%) [39] and Germany (6%) [13].

While the most commonly used drugs in the 70s were antispasmodics, benzodiazepines were most prevalent in the 80s, and antidepressants in the 90s. The annual prevalence of benzodiazepine use dropped from 21.6% in 1976 [4] to 9.3% in the 80s [1] 3% in 2002 [5] and to 3.1% in 2007 and antidepressants rose from 0.5% in 1976 [4], 0.3% in 1989 [1], 2% in 2002 [5] to 4.2% in 2007 in the city of São Paulo. The stabilization in psychotropic drug consumption in Brazil differs from results found in other countries such as the US, where an increase in consumption over the years has been observed [10, 12]. In the U.S. the use of antidepressants increased from 45% in 1987 to 79% in 1997, while benzodiazepine use decreased from 16% to 10% [11]. It is likely that the reduction in benzodiazepine use was a consequence of the introduction of new antidepressants, as well as the introduction of regulatory laws that limited the sale of benzodiazepines and barbiturates in the 80s.

A frequent point of contention among researchers is whether there has been an increase in the prevalence of depression/anxiety disorders or if the prevalence has always been high, just not detected. Depression prevalence, in São Paulo, ranged from 1% in the 80s [40], 7% in the 90s [41], 10% in 2000 [42] and decreased to 8% in 2007 [43]. For anxiety disorders, the increase in prevalence is even more significant, rising from 7% in the 80s [40] to 21% in 2007 [43]. Several factors may have contributed to an increase in the identification of cases in the community, both by individuals and by health professionals, such as better-defined diagnostic classifications [44], campaigns aimed at reducing stigma, and greater access to information about mental illness.

We observed a significant increase in the amount of prescriptions for psychotropic drugs by psychiatrists during the last 30 decades in São Paulo, from 11.7% in 1980 [1] to 41% in 2007. This change could be due to an increase in the number of psychiatrists in both the public and private systems, but this may also be a phenomenon specific to São Paulo, where formal employment is higher than in other regions of the country. In this study, we found that employment in São Paulo reaches 59.2%. Individuals who are formally employed usually have access to a health plan, which in turn facilitates access to a specialist [45]. These changes surely result in an improvement in individuals’ quality of life [46], as well as in the Brazilian health system [32], which also facilitates access to health services.

Our results confirm the public health policy of the free distribution of tricyclic inhibitors. Tricyclic users mostly obtained the medication by the government, while selective serotonin reuptake inhibitor (ISRR), selective norepinephrine reuptake inhibitor (SNRI) users and others bought the drugs from its own resources. Tricyclic are effective as inhibitors and other in the treatment of depression and anxiety [47], but cause significant side effects and are associated to Coronary heart disease [48, 49]. Moreover, despite increased access to free medication and the fact that these are most commonly prescribed by psychiatrists, the treatment gap remains high. Most individuals diagnosed with moderate/severe depression (67.5%) for which the use of antidepressants is indicated where not taking any psychotropic medications. These results agree with the national and international literature, which reports a treatment gap of 84% in Brazil [5], and 60% [50] to 90% [6] among individuals diagnosed with mental disorders in different studies in Europe. According to Andrade et al, [18] the main impediment to seeking treatment in moderate and mild cases is a attitudinal barrier. Whereas depression and anxiety (at different levels) are the most prevalent psychiatric disorders in the general population, greater availability of antidepressants that cause fewer side effects on the public health service, such as ISSR [51] could promote greater treatment adherence and consequently more effective recovery of the individual.

In São Paulo, an individual’s main access to treatment is primarily through the Estratégia de Saúde da Família (ESF; Family Health Strategy Program), introduced in 1996 ESF teams are multidisciplinary and include a general practitioner, a nurse, a community worker and a dentist, among others. Teams are trained to detect the most common problems affecting the population, and more specific psychiatric problems are delegated to psychiatrists and psychologists [52]. Considering the high prevalence of mental disorders in the general population [43, 53], these professionals should receive excellent training for recognizing and treating mental disorders. In this way, milder cases could be identified and treated adequately in primary care without being relegated to secondary and tertiary care.

Some limitations in this study should be considered: a) only diagnoses of depressive disorders, phobic disorders/anxiety and alcoholism were investigated; b) diagnoses were conducted using a standardized instrument with all the inherent limitations of the method; c) bias in remembering drugs used within the previous year, or mistakes with the commercial names or even not knowing that the medication used was a psychotropic drug; d) individuals might have received a prescription and not followed treatment; e) the diagnosis does not require the use of medication; disorders with milder/moderate symptoms do not necessarily have to be treated with a psychotropic drug, and these would be false positive cases in the treatment gap; and f) the use of homeopathic drugs or herbal medicines that are often used as tranquilizers was not included in this study. Another limitation was that we did not investigate certain details regarding the treatment, for example, the number of consultations made, or the dose or timing of medication use. This information would have enriched the discussion about the treatment gap.

## Conclusion

During 2007, 6% of the persons aged 15–75 in São Paulo were using a psychotropic drug in the past month; this was not a significant change from earlier years. We did not find that income was a major factor affecting the consumption of psychotropic drugs in this city, but there is a tendency of higher use with higher income. This could be a reflection of the government policy of free psychotropic distribution. Most people presenting a psychiatric illness during the previous month on the CIDI (85%) were not receiving any sort of medication, this gap may be due to a lack of identification of psychiatric cases in primary care units which could be improved if professionals receive further training in mental health.
